# Brittle Culm 12, a dual-targeting kinesin-4 protein, controls cell-cycle progression and wall properties in rice

**DOI:** 10.1111/j.1365-313X.2010.04238.x

**Published:** 2010-05-26

**Authors:** Mu Zhang, Baocai Zhang, Qian Qian, Yanchun Yu, Rui Li, Junwen Zhang, Xiangling Liu, Dali Zeng, Jiayang Li, Yihua Zhou

**Affiliations:** 1 State Key Laboratory of Plant Genomics and National Center for Plant Gene Research, Institute of Genetics and Developmental Biology, Chinese Academy of SciencesBeijing 100101, China; 2 State Key Laboratory of Rice Biology, China National Rice Research Institute, Chinese Academy of Agricultural SciencesHangzhou 310006, China

**Keywords:** kinesin-4 protein, cell cycle, microtubules, cellulose microfibrils, rice

## Abstract

Kinesins are encoded by a large gene family involved in many basic processes of plant development. However, the number of functionally identified kinesins in rice is very limited. Here, we report the functional characterization of *Brittle Culm12* (*BC12*), a gene encoding a kinesin-4 protein. *bc12* mutants display dwarfism resulting from a significant reduction in cell number and brittleness due to an alteration in cellulose microfibril orientation and wall composition. *BC12* is expressed mainly in tissues undergoing cell division and secondary wall thickening. *In vitro* biochemical analyses verified BC12 as an authentic motor protein. This protein was present in both the nucleus and cytoplasm and associated with microtubule arrays during cell division. Mitotic microtubule array comparison, flow cytometric analysis and expression assays of cyclin-dependent kinase (CDK) complexes in root-tip cells showed that cell-cycle progression is affected in *bc12* mutants. BC12 is very probably regulated by CDKA;3 based on yeast two-hybrid and microarray data. Therefore, BC12 functions as a dual-targeting kinesin protein and is implicated in cell-cycle progression, cellulose microfibril deposition and wall composition in the monocot plant rice.

## Introduction

Kinesins are ATP-driven microtubule-based motor proteins found in all eukaryotic organisms. The first kinesin was identified in squid giant axons as a protein involved in vesicle transportation (Brady, 1985; Vale *et al.*, 1985). Since then, evidence has suggested that microtubule-based motility, driven by kinesins, has diverse functions in many growth and developmental processes (Smith, 2003), including transport of organelles and molecules, control of microtubule dynamics and signal transduction, and direct or indirect involvement in cell division. It is estimated that approximately 0.1–0.13% of human genes encode kinesins, whereas *Saccharomyces cerevisiae* has only six, the fewest of all of sequenced organisms. Among eukaryotes, flowering plants have the highest number of *kinesin* genes. For example, Arabidopsis has 61, representing 0.24% of all Arabidopsis genes (Reddy and Day, 2001; Vale, 2003). Such abundance in the genome fits with the view that, in the absence of the microtubule organizing centers found in animals, plants require a higher number of motor proteins to facilitate a great diversity of microtubule configurations. The kinesins are classified into 14 subfamilies based on the conserved motor domain (Lawrence *et al.*, 2004), although the sequence outside the motor domain often shows low similarity. Therefore, grouping of certain kinesins into the same subfamily does not necessarily mean that they share similar functions (Lee and Liu, 2004).

Microtubules are present as a cortical array at interphase in plant cells. They are highly dynamic during cell morphogenesis (Lloyd, 1994), during which some microtubule-based motors participate in the (re)organization and (de)polymerization of microtubules. Mutation of these *kinesin* genes therefore results in disorganized cortical microtubules and abnormal cell shape. Perturbation of AtKIN5c, a kinesin localized to cortical microtubules, causes a root-swelling phenotype (Bannigan *et al.*, 2007). Cell morphogenesis is tightly associated with the production and arrangement of cellulose, the most abundant biopolymer of the cell wall. It is believed that cellulose microfibrils are typically arranged in patterns corresponding to the orientation of cortical microtubules (Ledbetter and Porter, 1963). Direct evidence for this arrangement came from a study tracking labeled cellulose synthase complexes at the plasma membrane of live cells (Paredez *et al.*, 2006). More recently, a new microtubule-associated cellulose synthase compartment was identified in Arabidopsis, providing clear evidence that the cortical microtubule pattern serves as a spatial template for the movement of cellulose synthase complexes (Crowell *et al.*, 2009; Gutierrez *et al.*, 2009). Kinesin motor proteins are believed to play a critical role in the alignment process (Zhong *et al.*, 2002; Smith and Oppenheimer, 2005). For example, FRA1 was reported to function as a kinesin involved in cellulose microfibril orientation via cortical microtubules although its function has not been fully elucidated (Zhong *et al.*, 2002).

In higher plants, cell division is a fundamental physiological process. The complex structural changes in microtubule arrays during cell division also require the involvement of several subfamilies of kinesins. Arabidopsis PAKRP1/kinesin-12A and PAKRP1L/kinesin-12B play critical roles in cytokinesis during male gametogenesis (Lee *et al.*, 2007). Plants lacking Arabidopsis kinesin-14 (ATK5) are defective in forming the early spindle, indicating a role for ATK5 in the search for and capture of anti-parallel interpolar microtubules (Ambrose and Cyr, 2007). Moreover, it has been demonstrated that the activity of many kinesins is regulated by cyclin-dependent kinase (CDK) complexes, a key component of the machinery regulating the orderly progression of the cell cycle (Inze and De Veylder, 2006). In Arabidopsis, CDKA;1 is the primary kinase controlling entry into the S and M phases of the cell cycle (Reichheld *et al.*, 1999; Dissmeyer *et al.*, 2007). Three of four kinesin-5 members in Arabidopsis contain the CDKA;1 phosphorylation site(s) (Vanstraelen *et al.*, 2006a). KCA1 and KCA2, two Arabidopsis mitosis-associated kinesin-14 proteins, also have two CDKA;1 phosphorylation sites each; these sites were implicated in protein folding and dimerization (Vanstraelen *et al.*, 2004). However, there are still very few identified CDKA-activated kinesins in plants.

Most members of the kinesin-4 subfamily in animals contain putative nuclear localization signals (NLSs) and function in mitosis (Kwon *et al.*, 2004; Mazumdar *et al.*, 2004). Arabidopsis has three kinesin-4 proteins with high similarity to their animal homologs, one of which (At5g60930) is up-regulated during mitosis (Vanstraelen *et al.*, 2006a). FRA1, which is found in the cytoplasm, was the first functionally identified kinesin-4 member in plants. Although the *fra1* mutant shows no defects in cell division, it does exhibit reduced plant height and fragile fibers as a result of aberrant deposition of cellulose microfibrils in the cell wall (Zhong *et al.*, 2002).

Whether other plant members of this subfamily function similarly to FRA1 remains unknown. Here, we report the functional identification of BC12 as a kinesin-4 subfamily member in *Oryza sativa*. Perturbation of *BC12* produces defects in cell number/plant height and mechanical properties. Similar to the typical kinesin-4 in animals, BC12 has an NLS and is present in both the cytoplasm and the nucleus. Furthermore, BC12 decorates some microtubule arrays during cell division and interacts with CDKA;3, probably undergoing phosphorylation for the purposes of regulation. Thus, in addition to the control of cellulose microfibril deposition and wall modification, BC12 also contributes to cell-cycle progression, and thus appears to exert multiple roles in cell division and wall biogenesis in rice.

## Results

### The *bc12* mutation results in altered mechanical strength

**Figure 1:**
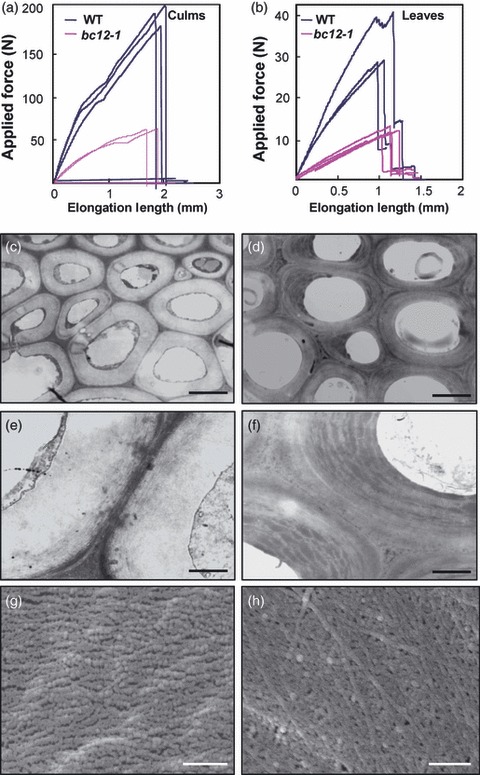
Mechanical properties of wild-type and *bc12* plants. (a) Measurements of the force required to break wild-type and *bc12-1* culms. (b) Measurements of the force required to break wild-type and *bc12-1* leaves. (c–f) TEM micrographs of the sclerenchyma walls of wild-type (c, e) and mutant (d, f) plants, showing the increase in electron-dense materials in the mutant walls. (g, h) Cellulose microfibrils in the innermost layer of wild-type (g) and *bc12-1* (h) sclerenchyma walls, showing a randomly oriented fiber pattern in *bc12-1* walls. Scale bars = 2 μm (c, d), 500 nm (e, f) and 250 nm (g, h).

A natural *brittle culm12* (*bc12*) mutant of the *japonica* cultivar *C418* showed reduced mechanical strength in culms and leaves. The breaking force of *bc12* culms and leaves was reduced to approximately 25% of that in the wild-type (Figure 1a,b), suggesting that the cell-wall composition may be altered in the mutant. We therefore compared the cellulose and lignin contents between *bc12* and wild-type culms. The cellulose content was not significantly altered, but the lignin content was increased by approximately 50% in *bc1*2 (Table 1). The higher lignin content resulted from a general increase in all three monomers (Table 1). Neutral sugar analysis revealed that the glucose content was not significantly altered (Table 2), but the content of both xylose and arabinose, two major sugars that form arabinoxylan, was increased by approximately 40% in *bc12*. However, the ratio of arabinose to xylose was not altered, indicating that *bc12* mutation causes an increase in the amount of arabinoxylan without affecting its structure.

**Table 1:** Cellulose and lignin content in wild-type and *bc12* culms

			Lignin monomer composition (μmol g^−1^ AIR)		
			
Samples	Cellulose content	Lignin content	G monomer	H monomer	S monomer
Wild-type	335.3 ± 2.3	89.8 ± 8.0	2.7 ± 0.2	10.8 ± 1.5	1.4 ± 0.2
*bc12-1*	342.0 ± 4.7	138.2 ± 9.1	3.9 ± 0.2	16.8 ± 2.7	1.6 ± 0.2

Alcohol-insoluble residues (AIR) prepared from the 2nd internodes of *bc12-1* and wild-type plants were used for compositional analysis.

The cellulose and lignin contents are given as mg g^−1^ AIR. Values are means ± SE of three independent assays.

**Table 2:** Neutral monosaccharide composition in wild-type and *bc12* culms

Samples	Rhamnose	Fucose	Arabinose	Xylose	Mannose	Glucose	Galactose
Wild-type	1.2 ± 0.4	0.5 ± 0.1	5.9 ± 0.6	39.5 ± 4.4	0.3 ± 0.1	331.7 ± 23.7	1.2 ± 0.2
*bc12-1*	1.2 ± 0.4	0.7 ± 0.1	8.0 ± 1.0	54.1 ± 3.9	0.3 ± 0.1	320.6 ± 30.5	1.3 ± 0.3

Alcohol-insoluble residues (AIRs) extracted from the 2nd internodes of wild-type and *bc12-1* plants were used for preparation of alditol acetates (see Experimental procedures). The glycosyl residues were quantified by GC/MS. Values are means ± SE (mg g^−1^ AIR) of three independent assays.

Mechanical strength is determined mainly by the properties of the secondary cell wall. Transmission electron microscopy showed that the wall thickness of sclerenchyma cells was not changed in *bc12* compared to the wild-type (Figure 1c,e). However, we found an increase in electron-dense materials in the mutant secondary walls, indicating structural abnormality in *bc12* plants (Figure 1d,f). We further visualized the cellulose microfibril pattern in the innermost secondary walls using field emission scanning electron microscopy (FESEM). The wild-type fibers were packed in a parallel pattern (Figure 1g), but those of the mutant plants were arranged in a random manner (Figure 1h). Taken together, the results show that the inferior mechanical strength of *bc12* is probably caused by the altered wall composition and aberrantly deposited cellulose microfibrils in the secondary walls.

### *bc12* plants have reduced plant height

**Figure 2:**
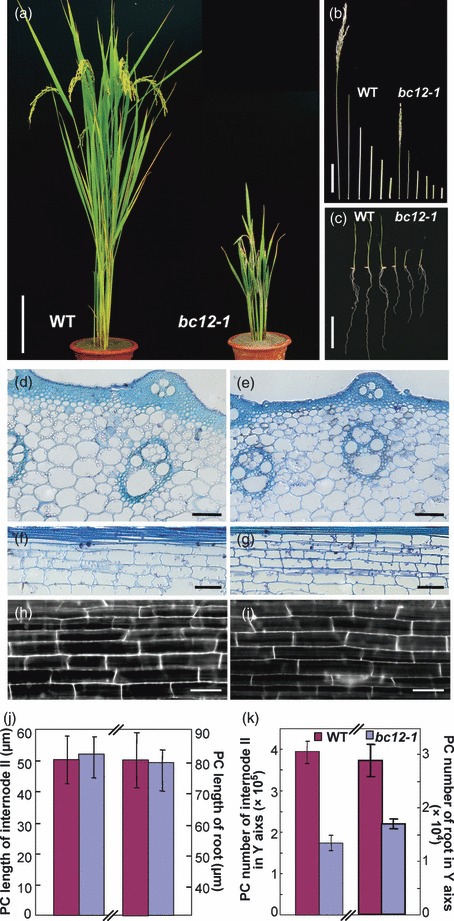
Phenotypic characterization of wild-type and *bc12* plants. (a) A wild-type and *bc12-1* plant. (b) Internodes of a wild-type and *bc12-1* plant. (c) Fourteen-day-old seedlings of wild-type and *bc12-1*. (d–g) Cross- and longitudinal sections of internode II of wild-type (d, f) and *bc12-1* (e, g). (h, i) Longitudinal views of epidermal cells in the mature region of wild-type (h) and *bc12-1* (i) roots of 7-day-old seedlings. (j) Length of parenchyma cells (PC) in internode II and in the mature root region of wild-type and *bc12-1*. Values are means ± SD (*n* = 5). (k) Number of parenchyma cells (PC) for internode II and roots at longitudinal direction of wild-type and *bc12-1*. Values are means ± SD (*n* = 5). Scale bars = 15 cm (a), 10 cm (b), 0.4 cm (c) and 50 μm (d–i).

Another major phenotype of *bc12* is severe dwarfism at all stages of growth and development. At the mature stage, the mutant plants were reduced in height by more than 50% compared to wild-type plants (Figure 2a) as a result of evenly shortened internodes in the mutant culms (Figure 2b). Additionally, the root length of 14-day-old mutant seedlings was only 60% of the wild-type root length (Figure 2c). To determine the reason for the dwarf phenotype in *bc12* plants, we examined the anatomical features of cells in the mutant and wild-type culms and roots. Culm cross-sections showed that the cell size of parenchyma and sclerenchyma cells was not significantly altered (Figure 2d,e), and the cell length observed in the longitudinal direction of culms and roots was similarly unchanged (Figure 2f–j), indicating that the decreased plant height and root length in *bc12* are not caused by a reduction in cell length or size. However, the total numbers of parenchyma cells in the longitudinal direction of the mutant culms (internode II) and roots were only 45 and 58%, respectively, of the wild-type numbers (Figure 2k). Therefore, the dwarf phenotype of *bc12* results from a reduced cell number.

### Map-based cloning of *BC12*

**Figure 3:**
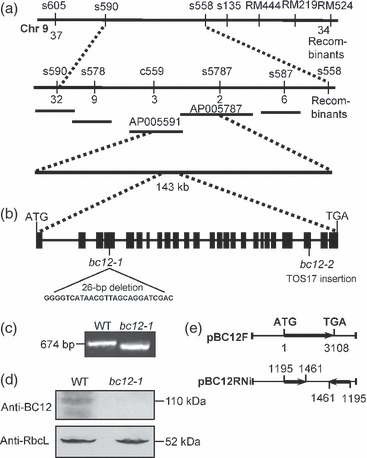
Map-based cloning and identification of *BC12*. (a) The *bc12* locus was mapped to a 143 kb region on chromosome 9. (b) The *BC12* gene. Black boxes indicate exons. Another allele of *bc12* is shown at the site where the mutation occurs. (c) The 26 bp deletion in *bc12-1* is demonstrated by comparing the amplified genomic DNA size between *bc12-1* and wild-type. (d) Protein gel blotting of total proteins isolated from wild-type and *bc12-1* seedlings using antibodies against BC12 and the Rubisco large subunit (RbcL). (e) Complementary construct (pBC12F) containing the full-length cDNA of *BC12* used for transforming *bc12* plants, and the knockdown construct (pBC12RNi) containing forward- and reverse-oriented cDNA fragments of *BC12* for transforming wild-type plants.

We used a map-based cloning approach to isolate the *BC12* gene. A total of 2056 F_2_ mutant plants were generated by crossing the mutant with *93-11*, a wild-type polymorphic *indica* variety. Genetic analysis placed the *BC12* locus between molecular markers s590 and s558 on chromosome 9, and the location of *BC12* was further refined to a 143 kb DNA segment covered by two BAC clones, AP005591 and AP005787 (Figure 3a), using the molecular markers described in Table S1.

Ten putative open reading frames (ORFs) are annotated by TIGR Rice Genome Annotation Project (http://rice.plantbiology.msu.edu) in the 143 kb DNA region. We sequenced and compared these with those of the wild-type. A 26 bp deletion was found in the 4th exon of one ORF, Os09g02650 (Figure 3b,c), resulting in a frameshift. Protein gel blotting detected no translational product using anti-BC12 antibodies raised in rabbit against a polypeptide corresponding to amino acid residues 697–900 (Figure 3d). An antibody against the Rubisco large subunit (RbcL) was used as the loading control. To demonstrate that *Os09g02650* corresponds to the *bc12* locus, we sequenced it in another allele *bc12-2*. The *bc12-2* allele harbors a TOS17 insertion in exon 23 (Figure 3b). To test for complementation, we generated transgenic rice plants by introducing the constructs shown in Figure 3(e) into the mutant and wild-type backgrounds. Over-expression of *BC12* cDNA in mutant lines rescued the mutant phenotypes, including both plant height and mechanical strength (Figure S1a–g). Protein gel blotting using anti-BC12 polyclonal antibodies showed that the complemented plants had an enhanced level of BC12 protein (Figure S1h). The knockdown lines mimicked the dwarfism and brittleness phenotypes of *bc12* (Figure S2a–g). The molecular basis of the suppression lines and *bc12-2* was confirmed by the low level and reduced size of *bc12* protein, respectively, as revealed by Western blotting (Figure S2h). Therefore, we have successfully cloned *BC12*.

### *BC12* is expressed mainly in organs undergoing cell division and secondary wall thickening

**Figure 4:**
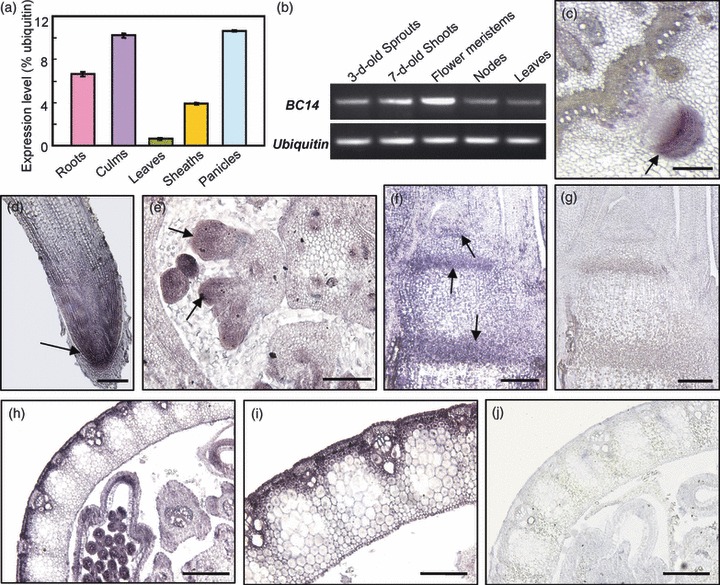
Expression pattern of *BC12*. (a) Expression of *BC12* in various organs. The expression levels are percentages of that of *Ubiquitin*. Values are means ± SD of triplicate assays. (b) RT-PCR amplification of *BC12* in tissues enriched in dividing and non-dividing cells, using the *Ubiquitin* gene as an internal control. (c–j) RNA *in situ* hybridization of *BC12* in wild-type plants. (c) Cross-section of a young stem, showing the signals in an initiating root. (d) Longitudinal section of a primary root. (e) Cross-section of a young culm at the flower developing stage. (f) Longitudinal section of a young stem. (h, i) Cross-section of a young culm at the heading stage (h) and a magnified image thereof (i). (g, j) Background controls, probed with a sense probe. Arrows indicate the apical or intercalary meristems. Scale bars = 70 μm (c, e–g, i), 200 μm (d) and140 μm (h, j).

Quantitative PCR revealed that *BC12* is universally expressed in all organs examined, with higher expression in panicles and culms (Figure 4a). RNA *in situ* hybridization further revealed the expression pattern of *BC12* at the tissue level. As shown in Figure 4(c–g), *BC12* is expressed in young tissues, including the initiating adventitious roots (Figure 4c), primary root tips (Figure 4d), flower primordia (Figure 4e) and intercalary meristems (Figure 4f), all tissues in which cells are undergoing vigorous cell division. This conclusion was confirmed by RT-PCR results showing greater expression of *BC12* in the tissues enriched in dividing cells than in those enriched in non-dividing cells (Figure 4b). Moreover, consistent with the quantitative PCR result that *BC12* is expressed in culms, hybridization signal was also detected in the sub-epidermal regions of young culms where sclerenchyma cells differentiate (Figure 4h–j). This expression pattern fits with the defects in cell number and wall properties observed in *bc12* plants.

### *BC12* encodes a kinesin-4 protein

**Figure 5:**
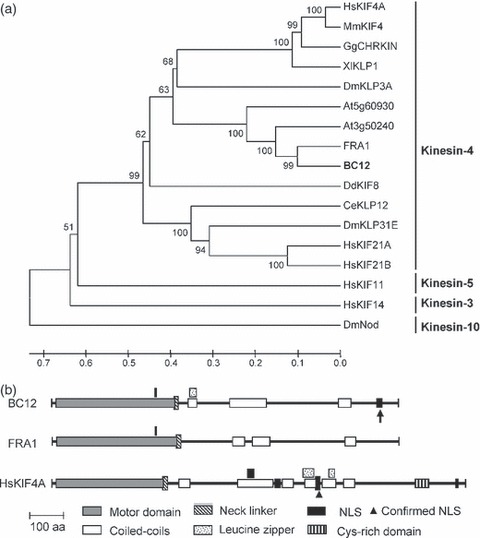
Phylogenetic and structure analyses of BC12. (a) Phylogenetic tree of BC12 and representative homologs from Arabidopsis and animals. The numbers at each node represent the bootstrap support (percentage), and the scale bar is an indicator of genetic distance based on branch length. (b) Schematic diagram of the domain structures of BC12, FRA1 and HsKIF4A. The overlaping domains are shown above where they are located. The nuclear localization signal (NLS) in BC12 is indicated by an arrow.

The ORF of *BC12* is 3108 nucleotides long and encodes a kinesin protein of the kinesin-4 subfamily, as revealed by a Pfam database search. A BLASTP search for BC12 homologs in rice and Arabidopsis genomes identified one and three kinesin-4 proteins harboring a complete motor domain, respectively. To determine the position of BC12 within the kinesin superfamily, an unrooted tree was built using the neighbor-joining method. Phylogenetic analysis revealed that BC12 and motor proteins selected from various kinesin subfamilies are divided into separated clades. Kinesin-4 proteins from several representative species were clustered together but formed different subclades. Among the kinesin-4 proteins, those from rice and Arabidopsis were found to belong to a monophyletic clade with 100% bootstrap support (Figure 5a). Interestingly, BC12 showed the closest homology to FRA1, which has been reported to be involved in cellulose microfibril deposition. We therefore compared the predicted domain structure among BC12, FRA1 and human KIF4A. Using several online servers (McDonnell *et al.*, 2006; Horton *et al.*, 2007; Finn *et al.*, 2008), we found common features among the three proteins, including a conserved motor domain at the N-terminus, a neck linker immediately after the motor domain, and a long coiled-coil region (Figure 5b). However, differences were found in the leucine zipper domain, the NLS and the C-terminal tail (Figure 5b). Although BC12 shows greater similarity to FRA1 than to HsKIF4A, one of the most important structural differences between BC12 and FRA1 is the possession of an NLS at the C-terminus of BC12, suggesting that it may perform functions distinct from those of Arabidopsis FRA1.

### BC12 shows microtubule-dependent ATPase activity

**Figure 6:**
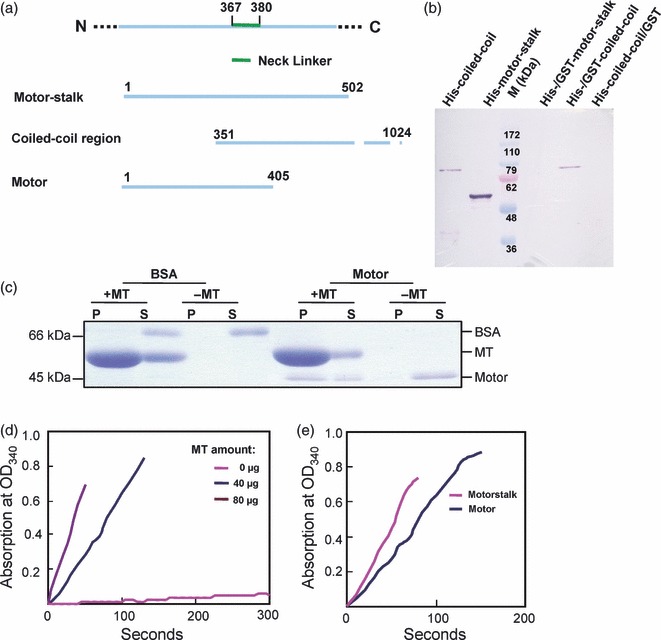
Biochemical properties of BC12. (a) Constructs for characterization of BC12 biochemical features. Details of the constructs are given in the text. (b) Western blotting of the GST affinity-purified GST- and His-tagged versions of the motor or coiled-coil domains using anti-His antibody. (c) An SDS–PAGE gel stained with Coomassie Brilliant Blue to reveal co-sedimentation of the motor domain with (−) and without (+) microtubules (MT). P, pellet; S, supernatant. (d) Absorbance of the reactions at 340 nm to reveal the ATPase activity of the purified motor domain in the presence of various amounts of microtubule (MT). (e) Absorbance of the reactions at 340 nm to reveal the ATPase activity of the motor domain in the presence of 40 μg of microtubules.

Typical kinesins are often dimerized *in vivo*. As BC12 is a putative kinesin, we determined which domain is responsible for dimerization. The motor-stalk region (amino acids 1–502), containing the motor domain and neck linker, and the long coiled-coil region (amino acids 351–1024) of BC12 (Figure 6a) were fused to histidine (His) and glutathione *S*-transferase (GST) for *in vitro* purification and examination of dimerization. The His- and GST-tagged versions of the motor-stalk region and the coiled-coil region were incubated together. An interaction was detected between the coiled-coil regions but not between the motor-stalk domains by immunoblotting with anti-His antibody (Figure 6b). The result indicates that the coiled-coil region of BC12 is responsible for dimerization.

BC12 also has a conserved ATPase-driven motor domain that is required for binding to and moving along the microtubules. To investigate the properties of this motor domain, a recombinant protein consisting of the motor domain (amino acids 1–405) (Figure 6a) was purified and incubated with microtubules. The purified protein co-sedimented with microtubules, but the majority of the protein remained in the supernatant in the absence of microtubules (Figure 6c), indicating that the motor domain of BC12 binds microtubules. We further examined the ATPase activity of the recombinant motor domain with the stalk region (amino acids 1–502) or without it (amino acids 1–405). The enzymatic activity was proportional to the amount of microtubules (Figure 6d). The motor domain with the stalk region showed higher enzymatic activity than the motor domain alone in the presence of 40 μg of microtubules (Figure 6e), suggesting that BC12 has microtubule-dependent ATPase activity and that the neck linker is critical for this activity.

### BC12 is present in both the nucleus and the cytoplasm

**Figure 7:**
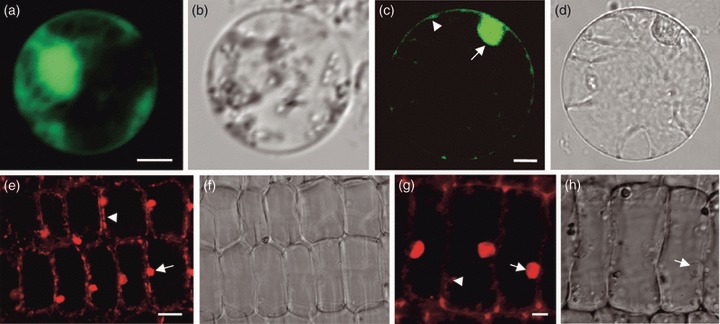
Subcellular localization of BC12. (a,b) Rice protoplast cell expressing GFP alone (a), showing fluorescent signals in the nucleus, the membrane and the cytoplasm. (b) DIC image. (c,d) Rice protoplast cell expressing BC12–GFP (c), showing signal in the nucleus (arrow) and in the cortex region of the cytoplasm (arrowhead). (d) DIC image. (e–h) Culm cells probed with anti-BC12 polyclonal antibodies (e), and magnified images (g), showing signals in the nucleus (arrows) and the periphery of cytoplasm (arrowheads). (f, h) DIC images. Scale bars = 15 μm (a–d) and 5 μm (e–h).

To determine the subcellular location of BC12, we expressed BC12–GFP in rice protoplasts and found that, in contrast to protoplasts expressing GFP alone (Figure 7a,b), the fluorescent signal of BC12–GFP was targeted to the periphery of transformed protoplast cells and to the nucleus (Figure 7c,d). Immunochemical staining of the rice culms using anti-BC12 polyclonal antibodies (Figure 7e–h) confirmed this localization. The nuclear localization suggests that BC12 contains an NLS, in line with the prediction from the domain analysis (Figure 5b). A 17 amino acid fragment (amino acids 971–987) was identified as the putative NLS based on bioinformatic analyses, and its role was experimentally investigated in a rat insulinoma cell line (INS-1) (Figure S3). INS cells expressing either the NLSn–GFP or NLSc–GFP, which include the NLS and its N- or C-terminal flanking sequence, respectively, showed abundant GFP signal in the nucleus (Figure S3a,d,e), in contrast to those transfected with GFP alone (Figure S3b), but identical to those transfected with BC12–GFP (Figure S3c). INS cells transfected with an NLS mutated at four positions displayed altered subcellular patterns (Figure S3a,f–i), indicating that the 17 amino acid sequence in BC12 is a functional NLS.

### BC12 localizes to mitotic arrays and the cytoplasm of dividing cells

**Figure 8:**
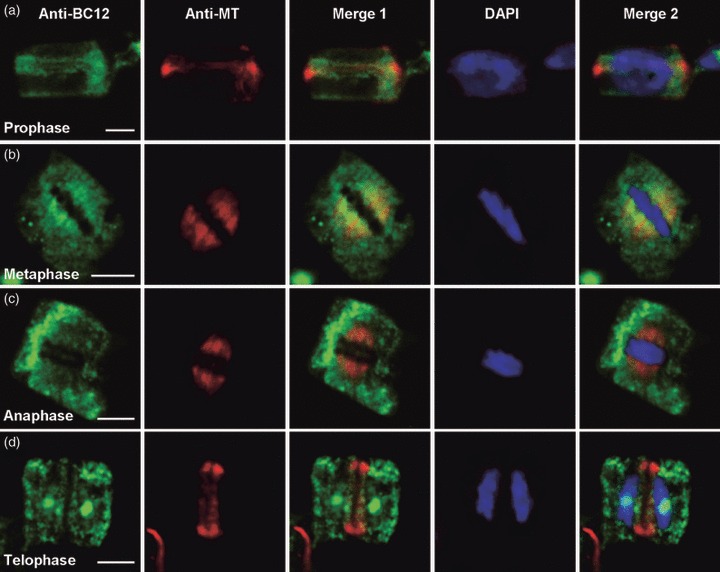
Immunolabeling BC12 and identical microtubule (MT) arrays in root-tip cells. (a) Prophase array. BC12 is detected along the preprophase band microtubules. (b) Metaphase array. Abundant BC12 signal is present in the cytoplasm and along kinetochore fibers. (c, d) Anaphase and telophase arrays. BC12 is shown in the cytoplasm and the nucleus. Merge 1 is a merged image of BC12 and microtubules. The cells were also stained with DAPI to detect DNA. Merge 2 is a merged image of BC12, microtubules and DAPI. Scale bars = 5 μm (a–d).

Both the nuclear localization of BC12 and the reduced cell number in *bc12* mutants suggest that BC12 may be involved in the cell cycle. We therefore examined whether BC12 localizes to mitotic microtubule arrays in dividing cells. Root-tip cells were used in the localization experiments. At prophase, when the preprophase band appeared, BC12 protein was localized along preprophase band microtubules (Figure 8a). When the mitotic spindle was assembled at metaphase, BC12 was abundantly present in the cytoplasm and along kinetochore fibers (Figure 8b). During anaphase and telophase, the BC12 signal was present in the cytoplasm (Figure 8c,d). The nuclear localization of BC12 persisted until late prophase and occurred again in late telophase (Figure 8d). Therefore, BC12 is abundantly localized in the cytoplasm and also associates with some mitotic microtubule arrays in dividing cells. To clarify the role of BC12 in the cell cycle, we compared the mitotic microtubule arrays between *bc12* and wild-type root cells. Unfortunately, no significant structural alteration was observed (Figure S4).

### Cell-cycle progression is delayed in *bc12* plants

**Figure 9:**
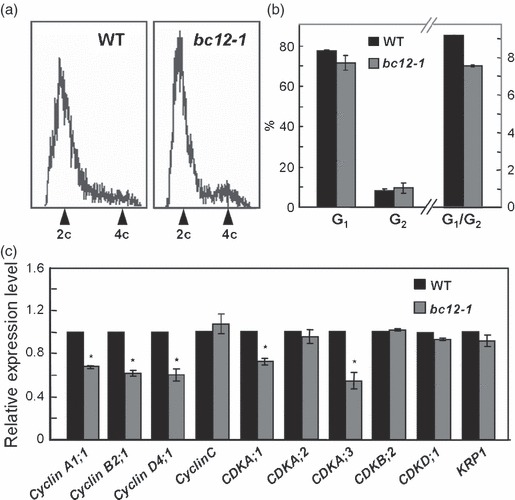
Cell-cycle progression is delayed in *bc12*. (a) DNA profiles of DAPI-stained wild-type and *bc12-1* root-tip nuclei measured in a flow cytometer. (b) Quantification of the DNA profiles in (a). (c) Relative expression levels of CDK complex genes in *bc12-1* and wild-type. The expression levels are given relative to that of wild-type. Asterisks indicate significance differences with respect to the wild-type (*t* test at *P* < 0.05).

To define the cell-cycle defects in *bc12*, we analyzed cell-cycle progression in root-tip cells of the mutant and wild-type plants by quantification of identical, immunolabeled microtubule arrays during the cell cycle. In mutant plants, the ratio of cells involved in mitosis (with the arrays of the preprophase band, spindle microtubule array and phragmoplast) to the total observed cells was only 38% of the wild-type ratio (Table 3). The cell cycle appeared to be retarded at interphase in *bc12*. Next, the DNA profiles of *bc12* and wild-type root-tip nuclei were measured using a flow cytometer. Mutant plants seemed to have fewer cells in G_1_ phase but more in G_2_ phase (Figure 9a), causing the ratio of G_1_ to G_2_ to be lower in mutant plants than in wild-type plants (Figure 9b). The decreased ratio of G_1_/G_2_ in *bc12* may result from the cell cycle being delayed at the G_2_ phase.

**Table 3:** Comparison of identical microtubule arrays during the cell cycle between *bc12* and wild-type root-tip cells

Sample	Cortical MT	Preprophase band	Spindle MT	Phragmoplast	Mitosis
*bc12-1*	15 099 (97.2)	158 (1)	85 (0.5)	176 (1.3)	419 (2.7)
Wild-type	5940 (92.9)	209 (3.3)	106 (1.7)	139 (2.2)	454 (7.1)

Values are the number of cells in which the MT array was observed, with percentages relative to the total number of cells observed given in parentheses. MT, microtubules.

The basic regulatory machinery governing the cell cycle at the key G_1_–S and G_2_–M transition points relies on CDK complexes (Inze and De Veylder, 2006). We therefore examined the expression levels of cyclin genes (*CYC*) and *CDK* genes in the mutant and wild-type using quantitative PCR. As shown in Figure 9(c), three *CYC* genes and two *CDKA* genes were down-regulated approximately 25–40% in *bc12* compared to the wild-type. Therefore, we conclude that the *bc12* mutation very likely delays cell-cycle progression at interphase, especially at the G_2_–M boundary.

### BC12 interacts with CDKA;3

**Figure 10:**
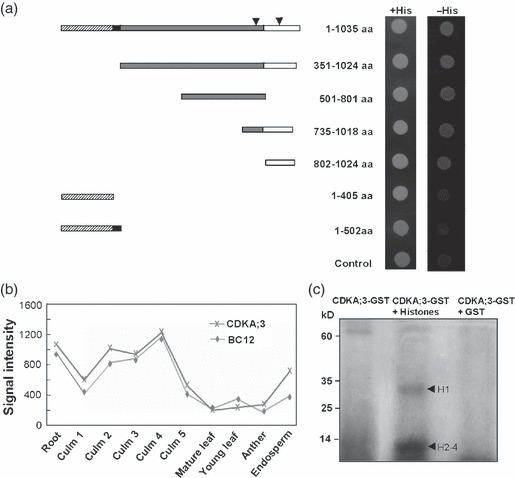
Interaction between BC12 and CDKA;3. (a) Identification of the BC12 interaction region with CDKA;3 by yeast two-hybrid assay. Details of the constructs and measurements are described in Experimental procedures. The arrowheads indicate the two phosphorylation sites on BC12. (b) Co-expression analysis of *BC12* and *CDKA;3* in various tissues of rice plants. Culms 1–5 represents every 5 cm from the top to the bottom of culms. (c) *In vitro* kinase assay of CDKA;3. Histones are mixture of H1–H4.

The above results prompted us to look for a direct link between BC12 and regulators of cell-cycle progression. At the C-terminus of BC12, two conserved CDKA phosphorylation sites (SPSK and SPPR) were found, suggesting that the activity of BC12 might be regulated by phosphorylation. To determine which CDK interacts with BC12, all of the 16 CDKs present in the rice genome were tested using a yeast two-hybrid assay. Of these, CDKA;3 showed an interaction with BC12 and BC12 fragments that contain one or two phosphorylation site(s) (Figure 10a), but this interaction was not found with the other 15 CDKs (Figure S5). Interacting proteins are generally spatio-temporally co-expressed. We therefore examined the expression of *BC12* and *CDKA;3* in various organs based on microarray data available in Gene Expression Omnibus (GEO) datasets (http://www.ncbi.nlm.nih.gov/) (Walczak, 2003). *BC12* is expressed with timing similar to that of *CDKA;3*, suggesting that *BC12* and *CDKA;3* may be regulated as co-expressed genes (Figure 10b). Moreover, a kinase assay showed that the CDKA;3 cloned from the rice genome is an authentic kinase based on its role in phosphorylating general substrates (e.g. histones) *in vitro* (Figure 10c).

## Discussion

### *bc12* is a kinesin-4 mutant in rice

Kinesins are microtubule-based motor proteins that release energy via ATP hydrolysis and utilize the energy to move along the cytoskeleton in order to perform various basic functions during plant development (Walczak, 2003). These basic functions are related to the typical domain features of kinesins (Lee and Kim, 2003; Lee and Liu, 2004; Mazumdar and Misteli, 2005). Here, we revealed that BC12 shares common domains with kinesins. Biochemical data showing that the motor domain of BC12 binds microtubules and has microtubule-dependent ATP hydrolysis activity, and that its long coiled-coil domain is required for dimer formation, identified BC12 as an authentic kinesin.

Arabidopsis has a great number of kinesins (61 kinesins) (Reddy and Day, 2001). Based on the sequenced genomes of both *japonica* and *indica* sub-species, rice has been predicted to have a number comparable to that of Arabidopsis (Umeki *et al.*, 2006b). However, the plant kinesins (even in Arabidopsis) are far from being as adequately characterized as kinesins in animals (Reddy and Reddy, 2002; Lu *et al.*, 2005; Ambrose and Cyr, 2007; Bannigan *et al.*, 2007). The functionally and genetically identified rice kinesins are much fewer (Sazuka *et al.*, 2005): rice kinesin identification has been mostly limited to *in vitro* biochemical characterization (Umeki *et al.*, 2006a, b; Frey *et al.*, 2009). This study investigated a *kinesin-4* mutant never before reported in rice, and the comprehensive analysis of the effects of the *BC12* mutation will further our understanding of kinesin functions in the monocot plant rice.

### BC12 is a cell cycle-related kinesin and affects cell-cycle progression

Cell division is a fundamental process governing the central elements of plant growth and development. In Arabidopsis, at least one third of kinesins are involved in mitosis, according to expression datasets of synchronized cell cultures (Vanstraelen *et al.*, 2006a). Increasing genetic evidence has also revealed direct or indirect contributions of some plant kinesins to cell division (Lee *et al.*, 2001; Pan *et al.*, 2004; Ambrose and Cyr, 2007; Bannigan *et al.*, 2007). Since the first mitosis-related kinesin-4 was characterized in mice (Sekine *et al.*, 1994), various members of this subfamily in animals have been identified as chromokinesins (Mazumdar and Misteli, 2005). However, in plants, due to the lack of mutants, few kinesin-4 proteins have been functionally reported, and to date none has been shown to be involved in mitosis or the cell cycle.

Of the three kinesin-4 proteins in Arabidopsis, FRA1 was the first to be functionally documented. However, a role for FRA1 in cell division has not been observed (Zhong *et al.*, 2002). Although bioinformatic analysis places BC12 in a monophyletic clade with FRA1, it has several distinct features. The most prominent difference is the presence of an NLS in BC12. Therefore, in addition to the observation that mutation of *BC12* caused reduced cell number in culms and roots, the following findings suggest that BC12 is a cell cycle-related kinesin:

- *BC12* is highly expressed in organs undergoing cell division,
- its nuclear localization was experimentally verified by expressing GFP fused to wild-type or point-mutated NLSs in rice protoplasts and rat INS cells,
- BC12 localizes to microtubule arrays during cell division,
- the *bc12* mutation appeared to delay cell-cycle progression at interphase, especially at the G_2_–M boundary, as revealed by quantitative comparison of the distinct mitotic microtubule arrays and the DNA profiles in wild-type and *bc12* root-tip cells, and
- the expression level of CDK complex genes is reduced in the mutant plants.

CDK complexes are well-known regulators that aid cell-cycle progression by phosphorylation of downstream effectors (Morgan, 1997). CDKA phosphorylation sites exist in 14 of 23 mitosis-related Arabidopsis kinesins (Vanstraelen *et al.*, 2006a), such as kinesin-5 homologs (Vanstraelen *et al.*, 2006a) and KCA1, a member of the kinesin-14 subfamily in Arabidopsis (Kong and Hanley-Bowdoin, 2002). Unlike the Arabidopsis kinesin-4 members, which lack classic CDKA phosphorylation sites (Vanstraelen *et al.*, 2006a), BC12 contains two conserved CDKA phosphorylation sites, suggesting that it might be regulated by CDK complexes. Yeast two-hybrid screening revealed an interaction between CDKA;3 and BC12. Moreover, the C-terminus of BC12, containing the consensus phosphorylation site(s), contributes to this interaction. The co-expression pattern of *BC12* and *CDKA;3* further suggests that BC12 may be regulated by CDKA;3 in rice. We also explored whether BC12 could be phosphorylated by CDKA;3 *in vitro*. However, probably due to the lack of a properly joined cyclin and/or other components, phosphorylation activity was not detected (data not shown). Phosphorylation is an important means of regulation of kinesin activity during cell-cycle progression (Vanstraelen *et al.*, 2004). Studies have investigated how phosphorylation of KCA1 affects its dimerization, folding and cellular localization, thereby regulating its function in mitosis (Vanstraelen *et al.*, 2004, 2006b). BC12 may be a potential target of the authentic kinase CDKA;3. *bc12* mutation disrupts the CDKA phosphorylation sites, resulting in altered cell-cycle progression.

### BC12 functions in mechanical strength

The interphase microtubules just below the plasma membrane are believed to have the ability to direct the patterning of cellulose microfibrils in both the primary and secondary cell walls (Heath, 1974; Seagull, 1992). Organization of cortical microtubules is energy-dependent and probably involves some kinesins (Wymer *et al.*, 1996). Perturbation of these kinesins results in impaired morphogenesis and/or an abnormal cellulose deposition pattern (Reddy and Day, 2000; Reddy and Reddy, 2002). In this study, mutation of *BC12* caused abnormal deposition of cellulose microfibrils and decreased the mechanical strength of rice culms, indicating that cytoplasmic BC12 may play a role in cortical microtubule organization and/or cellulose microfibril deposition. However, consistent with our observation that cell size and cell shape in *bc12* and wild-type were indistinguishable, the organization of cortical microtubules in *bc12* is not significantly altered. Such contradictions have been reported in several other mutants. The *cobra* mutant that exhibits a loss of anisotropic growth in Arabidopsis roots has disorganized cellulose microfibrils (Schindelman *et al.*, 2001; Roudier *et al.*, 2005), but the orientation of cortical microtubules is not significantly affected in the root elongation zone (Hauser *et al.*, 1995). The Arabidopsis *fra1* mutants, in which the function of kinesin-4 has been destroyed, have an altered orientation of cellulose microfibrils despite normal cortical microtubules and unchanged cellulose and lignin content (Zhong *et al.*, 2002). Thus, the molecular mechanism by which cortical microtubules regulate the orientation of cellulose microfibrils is more complicated than one would expect. Further studies are required to unambiguously elucidate the functions of BC12 in micro-tubule organization and wall modification in rice plants.

### Mutation of *BC12* reveals a potential link between cell growth and cell-wall modification

Many mutants defective in cell-wall formation exhibit abnormal growth and morphogenesis (Pien *et al.*, 2001). One can thus speculate that the cell-wall biogenesis and modification are tightly associated with cell growth via key genes at the interface of morphogenesis, the cell cycle, and cell-wall biogenesis (Somerville *et al.*, 2004). Due to the limitations of available analytical approaches, few of these genes can be identified in a developmental context (Somerville *et al.*, 2004). Here, our study of a *bc12* mutant, as summarized by three key concepts, namely cell-cycle progression, wall modification and microtubules, indicates that BC12 is at the interface between the cell cycle and cell-wall biogenesis.

BC12 shows both cytoplasmic and nuclear localization patterns. *bc12* culms exhibit cell-wall abnormalities, including randomly oriented cellulose microfibrils, increased lignin and arabinoxylan contents, and altered sclerenchyma wall structure, indicating that cytoplasmic BC12 may directly or indirectly affect cellulose deposition and wall composition. Its presence in the nucleus at interphase and localization to mitotic microtubule arrays during cell division, as revealed in root-tip cells, suggest that BC12 function may not be restricted to cell-wall biogenesis, and that it is an essential kinesin involved in cell-cycle progression. The phenomenon whereby one motor protein functions both in the cell cycle and in wall modification has been observed very rarely in plants. The phenotype of the temperature-sensitive Arabidopsis mutant *radially swollen7* (*rsw7*), which was originally described as having a defect in root swelling, is probably caused by abnormal cell-wall composition (Wiedemeier *et al.*, 2002). *RSW7* was subsequently reported to encode AtKIN5c, one of the four Arabidopsis kinesin-5 members, and to play central roles in mitotic spindle architecture and cortical microtubule organization (Bannigan *et al.*, 2007).

In conclusion, the abnormal phenotypes with respect to cell number and wall properties in the *bc12* mutant suggest that BC12 may be a good subject for study in order to elucidate an interesting link between cell growth and cell-wall formation and for understanding kinesin functions in the monocot plant rice.

## Experimental procedures

### Plant materials

The rice (*Oryza sativa* L.) *brittle culm12* mutants (*bc12-1* and *bc12-2*) were isolated from *japonica* cultivar C418 and Nipponbare, respectively. A F_2_ mapping population was generated from the cross between *bc12-1* and *93-11*, a polymorphic *indica* cultivar. All the plants used in this research were cultivated in experimental fields at the Institute of Genetics and Developmental Biology (Beijing, China) and the China National Rice Research Institute (Hangzhou), or in Hainan province, during the natural growing season.

### Measurement of mechanical properties

The breaking force and extensibility of rice culms and leaves were measured using a digital force/length tester (5848 microtester; Instron, http://www.instron.com). To avoid inaccuracies, age-matched second internodes and flag leaves of the same width and length were used for immediate measurement. The breaking force was calculated as the force required to break apart a culm or leaf segment. The extension length was the distance that the tested samples extended before breaking.

### Cell wall analysis

Air-dried culms (1 g) of the second internodes were ground into a fine powder. Alcohol-insoluble residues of the walls were obtained by extracting the samples in 70% v/v ethanol at 70°C and drying in an oven at 80°C, and the residues prepared for the lignin assay were extracted using 80% v/v methanol. The alcohol-insoluble residues were hydrolyzed in 67% v/v H_2_SO_4_ for 1 h at room temperature, and then in 2 m H_2_SO_4_ at 121°C for 1 h. The alditol acetate derivatives were determined by GC/MS (Tanaka *et al.*, 2003). The crystalline cellulose was measured using a method as described by Updegraff (1969). The lignin content and those of its monomers were analyzed as described previously (Kirk and Obst, 1988; Hoebler *et al.*, 1989).

### Microscopy

Culm segments were fixed in 2.5% v/v glutaraldehyde in PBS (4 mm sodium phosphate, 200 mm NaCl, pH 7.2) at 4°C overnight. Samples were extensively washed in the same buffer, and post-fixed in 2% w/v OsO_4_ for 0.5 h. After dehydration in an ethanol series, the samples were infiltrated and embedded in butyl-methyl methacrylate. Sections 3 μm thick were cut, stained with toluidine blue, and viewed under a light microscope (Leica, http://www.leica-microsystems.com). The root cell length was determined by staining the whole roots of 7-day-old seedlings with 20 μg ml^−1^ of propidium iodide in PBS, followed by examination under a fluorescent microscope (Leica). For transmission electron microscopy, the samples were embedded in Spurr resin (Sigma-Aldrich, http://www.sigmaaldrich.com/), sectioned (80 nm thick) with an Ultracut E ultramicrotome (Leica), and picked up using Formvar-coated copper grids (SPI supplies, http://www.2spi.com). After post-staining with uranyl acetate and lead citrate, the specimens were viewed under a Hitachi H7500 transmission electron microscope (http://www.hitachi.com).

To view the alignment of cellulose microfibrils, culms from development-matched wild-type and *bc12-1* plants were sliced and fixed in 4% paraformaldehyde (Sigma). The samples were thoroughly rinsed in PBS buffer and treated with Updegraff reagent (Updegraff, 1969) at 100°C for 60 min to solubilize pectin, hemicellulose and non-crystallized cellulose. The samples were then washed in distilled water and dehydrated in an ethanol series for 30 min each. After critical-point drying using liquid CO_2_, all of the samples were mounted on double-sided sticky carbon tape with the cut surface facing upward, and then coated with platinum at 20 mA for 120 sec. The cell wall structure was examined using a JSM6700F field emission scanning electron microscope (JEOL, http://www.jeol.com).

### Map-based cloning

*BC12* was mapped and cloned using 2056 F_2_ mutant plants and the molecular markers listed in Table S1. The corresponding DNA fragments were amplified from the mutant and wild-type plants using LA-Taq (TaKaRa; http://www.takara.com) and sequenced using a ABI 3730 sequencer (Applied Biosystems, http://www.appliedbiosystems.com). To reveal the 26 bp deletion in *bc12-1*, genomic DNA fragments covering 2500–3174 bp after the start codon were amplified from *bc12-1* and wild-type plants.

For protein gel blotting, 1 g (fresh weight) of 7-day-old mutants, wild-type plants and transgenic plants were ground in liquid nitrogen and homogenized in extract buffer [25 mm Tris/HCl, pH 7.5, 0.25 m sucrose, 2 mm EDTA, 2 mm DTT, 15 mmβ-mercaptoethanol, 10% glycerol and proteinase inhibitor cocktail (Roche; http://www.roche.com). After centrifugation at 10 000 ***g*** for 20 min, the supernatant was electrophoresed by 10% SDS–PAGE, blotted onto a nitrocellulose membrane (Amersham; http://www.gelifesciences.com), and probed with antibodies against BC12 or the Rubisco large subunit (RbcL). Anti-BC12 polyclonal antibodies were generated in rabbit against the polypeptides comprising amino acids 697–900. BC12-specific IgG was further purified through affinity chromatography using recombinant BC12 fusion protein produced in *Escherichia coli* BL21 (DE3) as ligand.

For complementation testing, the full coding region of BC12 driven by the *actin* promoter was inserted into the binary vector pCAMBIA 1300 to generate the plasmid pBC12F. To knockdown *BC12*, the cDNA fragment, amplified by the primers listed in Table S2, was inserted into pKANNIBAL 1300 in both the forward and reverse orientation to generate the plasmid pBC12RNi. The two binary plasmids were introduced into *Agrobacterium tumefaciens* strain *EHA105* by electroporation and used for rice transformation.

### Gene expression analysis

Total RNA was extracted from 30-day-old leaves, leaf sheaths and roots, 60-day-old culms and panicles, 3-day-old sprouts, 7-day-old shoots and apical meristems at the flowering stage as described previously (Li *et al.*, 2003). cDNA was synthesized from total RNA using Promega reverse transcriptional kits (http://www.promega.com). The real-time PCR primers for amplification of *BC12* and *Ubiquitin* are shown in Table S2. The reactions were performed using a ABI 7900HT quantitative PCR system (Applied Biosystems).

RNA *in situ* hybridization was performed as described by Li *et al.* (2003). The 3′ end of *BC12* was subcloned into the pGEM-T Easy vector (Promega) and used as a template to generate RNA probes. Hybridization was performed on wax-embedded transverse sections (10 μm thick) using the probe labeled by a DIG RNA labeling Kit (Roche). The slides were observed under a light microscope (Leica) and photographed using a CCD camera.

### Biochemical properties of BC12

*BC12* cDNA fragments containing either the motor domain (amino acids 1–502) or the coiled-coil region (amino acids 351–1024) were fused in-frame with either histidine (His) in the pET28a expression vector or glutathione *S*-transferase (GST) in the pGEX6P-1 expression vector (Novagen; http://www.merck-chemicals.com). The fusion proteins were expressed in *E. coli* and purified for dimerization analysis. His- and GST-tagged motor or coiled-coil domains were incubated at 4°C for 5 h and purified by GST affinity chromatography. The eluate was used for protein gel blotting with anti-His monoclonal antibody (Sigma).

For the microtubule co-sedimentation assay, recombinant proteins containing the motor domain of BC12 with the stalk region (amino acids 1–502) and without it (amino acids 1–405) were purified by expressing them in vector pGEX6P-1. The microtubule co-sedimentation assay was performed as described previously (Zhong *et al.*, 2002). Tubulin subunits and the recombinant protein were visualized by staining the gel with Coomassie Brilliant Blue R250 (Sigma). BSA was used as a negative control. In addition, we used a Steady-State ATPase Assays Coupled Enzyme System (http://www.proweb.org/kinesin) to determine the ATP-hydrolyzing activity of the motor domain. Specifically, microtubules were prepared by incubation of 10 mg ml^−1^ tubulin (Cytoskeleton, http://www.cytoskeleton.com) in PEM buffer (80 mm Na/PIPES, pH 6.9, 1 mm MgCl_2_, 1 mm EGTA) containing 1 mm GTP and 10% glycerol at 37°C for 50 min. Microtubules were stabilized by adding an equal volume of PEM buffer containing 4 mm taxol, and then incubated at 37°C for 10 min. Reactions were performed by mixing 10 μg purified recombinant protein with various concentrations of prepared microtubules in PEM buffer containing 25 mm Tris-OAc, 0.5 mm MgCl_2_, 0.5 mm DTT, 1.5 mm Mg-ATP, 3 mm phosphoenolpyruvate, 0.225 mm NADH^+^ and 1.75% v/v pyruvate kinase/lactate dehydrogenase (Sigma). This solution was mixed quickly and placed into a spectrophotometer (Beckman, http://beckman-coulter.com). After 1 min, the absorbance at 340 nm was measured at 10 sec intervals for 300 sec.

### Subcellular localization analysis

To determine the exact subcellular localization of BC12, the *BC12* cDNA was fused in-frame with EGFP and inserted between the CaMV 35S promoter and the NOS terminator in the PUC19 vector. The expression construct was transfected into rice protoplasts, with EGFP alone as the control.

To confirm the BC12 localization in rice plants, affinity-purified anti-BC12 polyclonal antibodies were used. Fresh hand-cut sections of rice culms were fixed in 4% paraformaldehyde (Sigma) in PBS for 2 h. Samples were extensively washed with the same buffer and blocked in PBS containing 1% w/v BSA for 0.5 h. After rinsing three times in PBS containing 0.1% w/v BSA, the sections were incubated with purified BC12 antibodies at a 1:50 dilution at 4°C overnight. The secondary antibodies, Cy3-conjugated anti-rabbit IgG (Sigma), were added at a 1:500 dilution. Labeled sections were observed using a confocal microscope (LSM 510 META; Zeiss, http://www.zeiss.com).

### Identification of the NLS in BC12

To identify the NLS sequence of BC12, cDNA fragments containing wild-type and point-mutated NLSs (amino acids 971–987) were fused in-frame with EGFP in the pCDNA3.1 vector (Invitrogen, http://www.invitrogen.com). Then the plasmid DNAs for the above constructs and empty vector were purified and used in a modified lipofection procedure to transiently transfect INS cells. After incubation for 3 h, the infected cells were transferred to Opti-MEM medium (Invitrogen), cultured for 1–2 days, and observed using a confocal microscope (Olympus, http://www.olympus-global.com/).

### Cell cycle examination

To observe microtubule arrays during cell division, wild-type and *bc12-1* seeds were germinated on the wet filter paper for 3 days. Roots were then excised and fixed in 4% paraformaldehyde (Sigma) for 1 h and digested using 0.1% (w/v) pectolyase Y-23 and 1% (w/v) cellulase R10 (Yakult, http://www.yakult.co.jp) in PBS buffer at 37°C for 1 h. The root tips were squashed on clean glass slides, and the slides were used for immunochemical staining. The antibodies used and the final concentrations were as follows: affinity-purified anti-BC12 antibodies, 1:50 v/v; FITC-conjugated anti-rabbit IgG secondary antibody (Sigma), 1:100 v/v; anti-α-tubulin antibody (Sigma) 1:100 v/v; Cy3-conjugated anti-mouse IgG secondary antibody (Sigma), 1:100 v/v. The samples were stained with 2 μg ml^−1^ 4,6-diamidine-2-phenyl indole (DAPI; Sigma) and viewed under a confocal laser scanning microscope (TCS SP5; Leica).

Cell-cycle progression was examined by comparing the DNA profiles of wild-type and mutant root-tip cells. The nuclei of 2-day-old root tips were released as described by Galbraith *et al.* (1983). Specifically, root tips were cut and chopped in Galbraith's buffer (45 mm MgCl_2_, 30 mm sodium citrate, 20 mm 4-morpholinepropane sulfonate, 1 mg ml^−1^ Triton X-100, pH 7.0) using a razor blade. Then, the nuclei were filtered through a 300 mesh. After staining with DAPI (20 μg ml^−1^), the nuclei were subjected to flow cytometric analysis using a Cell Lab Quanta SC counter (Beckman Coulter). At least 10 000 nuclei were measured for each sample.

To detect expression of cell-cycle regulatory genes, total RNA were extracted from 7-day-old wild-type and *bc12-1* seedlings. Then cDNA was synthesized using an oligo(dT) primer (Promega). The primers used for quantitative PCR of cyclin/CDK complex genes are given in Table S2. The reactions were performed on an ABI 7900HT quantitative PCR system (Applied Biosystems).

### Yeast two-hybrid experiments

The BC12 fragments shown in Figure 10(a) were generated by PCR and ligated into the pGEM-T Easy vector (Promega). After sequencing confirmation, they were fused into the pDBLeu vector (Gibco BRL; http://www.invitrogen.com). CDKA;3 and the other 15 CDKs were cloned from rice and inserted in-frame into the pPC86 vector (Gibco BRL). Yeast strain MaV203 was co-transformed with pDBLeu-BC12x (where x indicates the various BC12 fragments) and pPC86-CDKA;3. After incubating on medium without Leu and Trp, co-transformants were spotted with an equal cell suspension on medium lacking His. The strength of the protein–protein interaction was measured by the ability to grow on His-free medium supplemented with 10 or 25 mm 3-amino-1,2,4-triazole (Sigma). As a negative control, the BC12 fragments were co-transformed with the empty vector (control) and spotted on the same medium.

### *In vitro* phosphorylation assay

The autophosphorylation assay was performed by incubating 1 μg of purified CDKA;3–GST fusion protein in reaction buffer (50 mm Tris/HCl, pH 7.6, 10 mm MgCl_2_, 1 mm DTT, 0.1 mm ATP) in the presence of 5 μCi [γ-32P]ATP at room temperature for 30 min. The reaction was stopped by addition of protein gel loading buffer. The substrate phosphorylation assay was performed by adding 5 μg histones. The phosphorylated products were separated by 12% SDS–PAGE and detected by autoradiography.

## SUPPORTING INFORMATION

**Figure S1.** Characterization of complementary transgenic plants.

**Figure S2.** Characterization of *BC12* suppression transgenic plants.

**Figure S3.** Identification of the NLS in BC12.

**Figure S4.** Comparison of microtubule arraysduring the cell cycle between the wild-type and *bc12-1* root cells.

**Figure S5.** Yeast two-hybrid assay of BC12 and CDKs from rice.

**Table S1.** Molecular markers developed for map-based cloning of *BC12*.

**Table S2.** Primer sequences used in this study.

Please note: As a service to our authors and readers, this journal provides supporting information supplied by the authors. Such materials are peer-reviewed and may be re-organized for online delivery, but are not copy-edited or typeset. Technical support issues arising from supporting information (other than missing files) should be addressed to the authors.
