# Supplementary figures and images for: Brittle Culm 12, a dual-targeting kinesin-4 protein, controls cell-cycle progression and wall properties in rice
Source: Plant J. 2010 May 26;63(2):312–28. doi: 10.1111/j.1365-313X.2010.04238.x (PMC3440585; doi:10.1111/j.1365-313X.2010.04238.x)

Figure S1

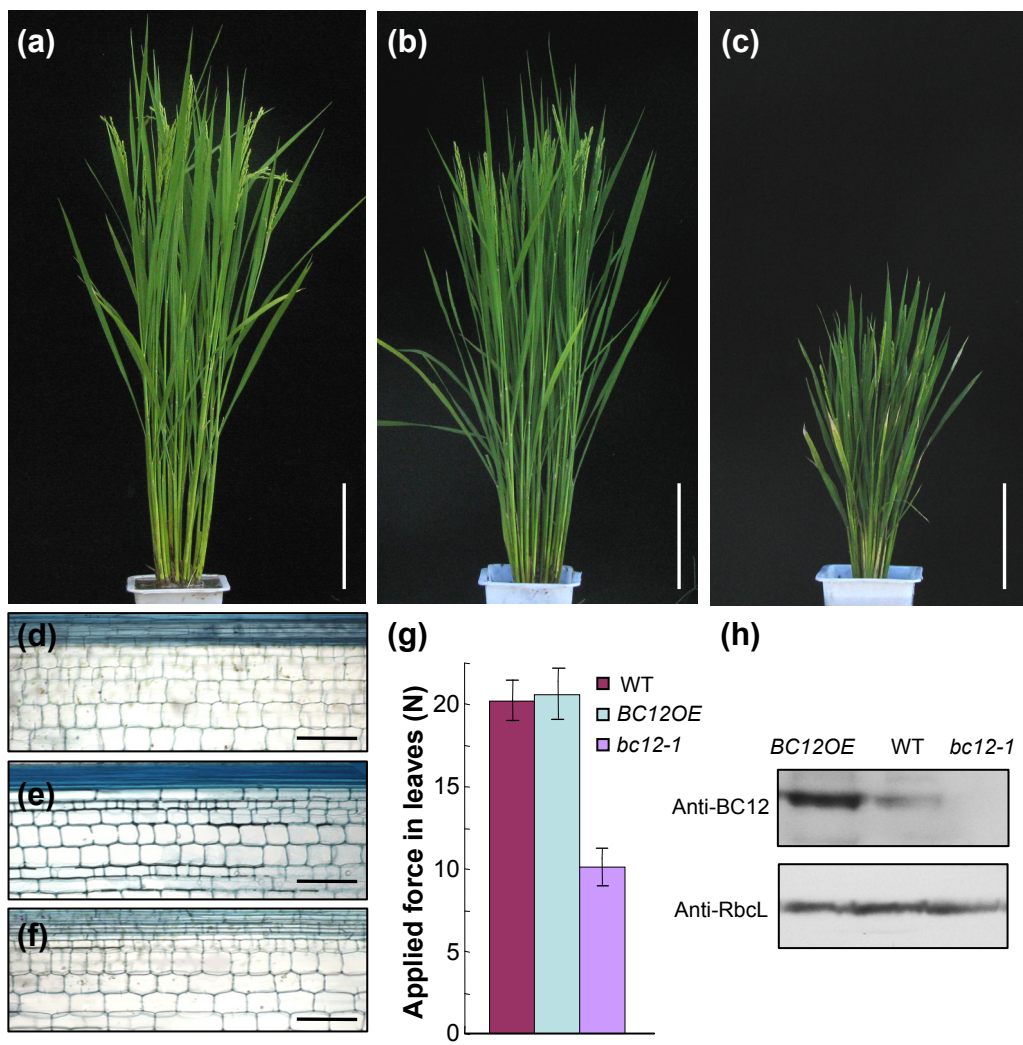

Figure S2

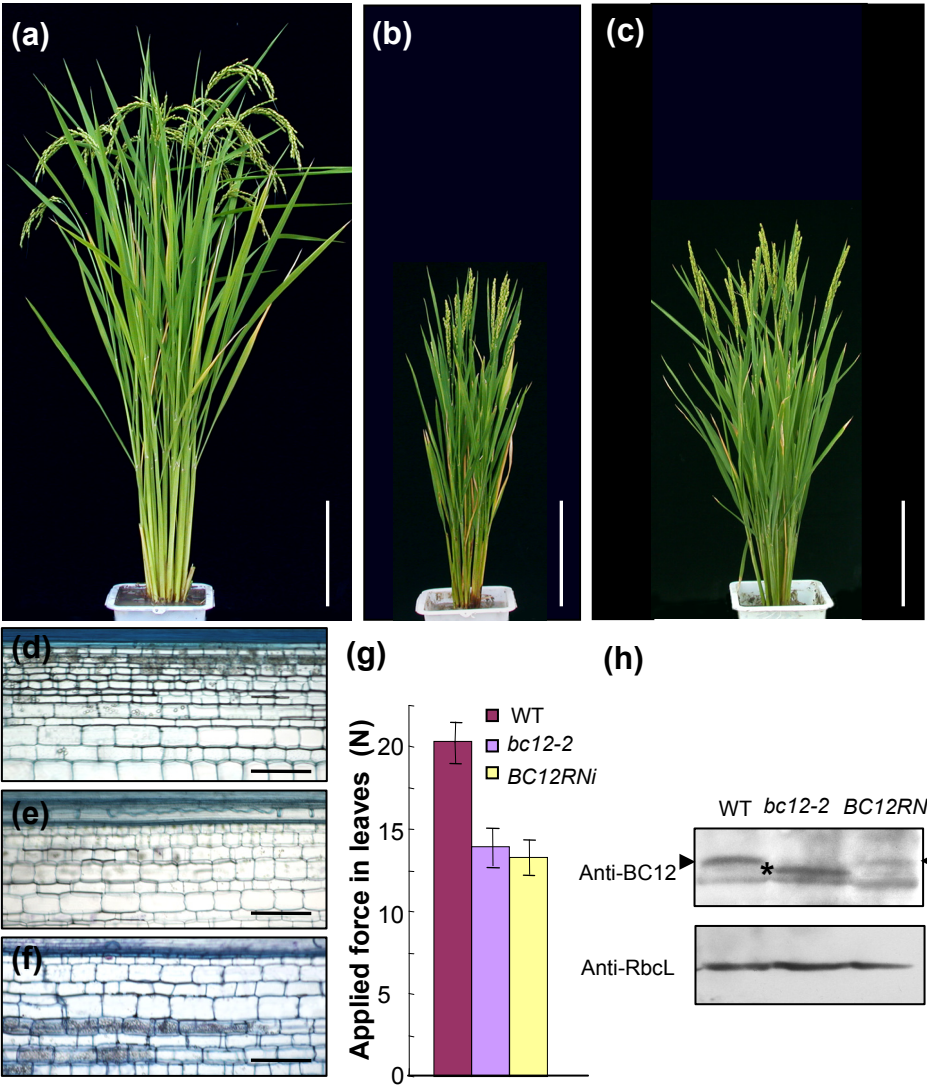

Figure S3

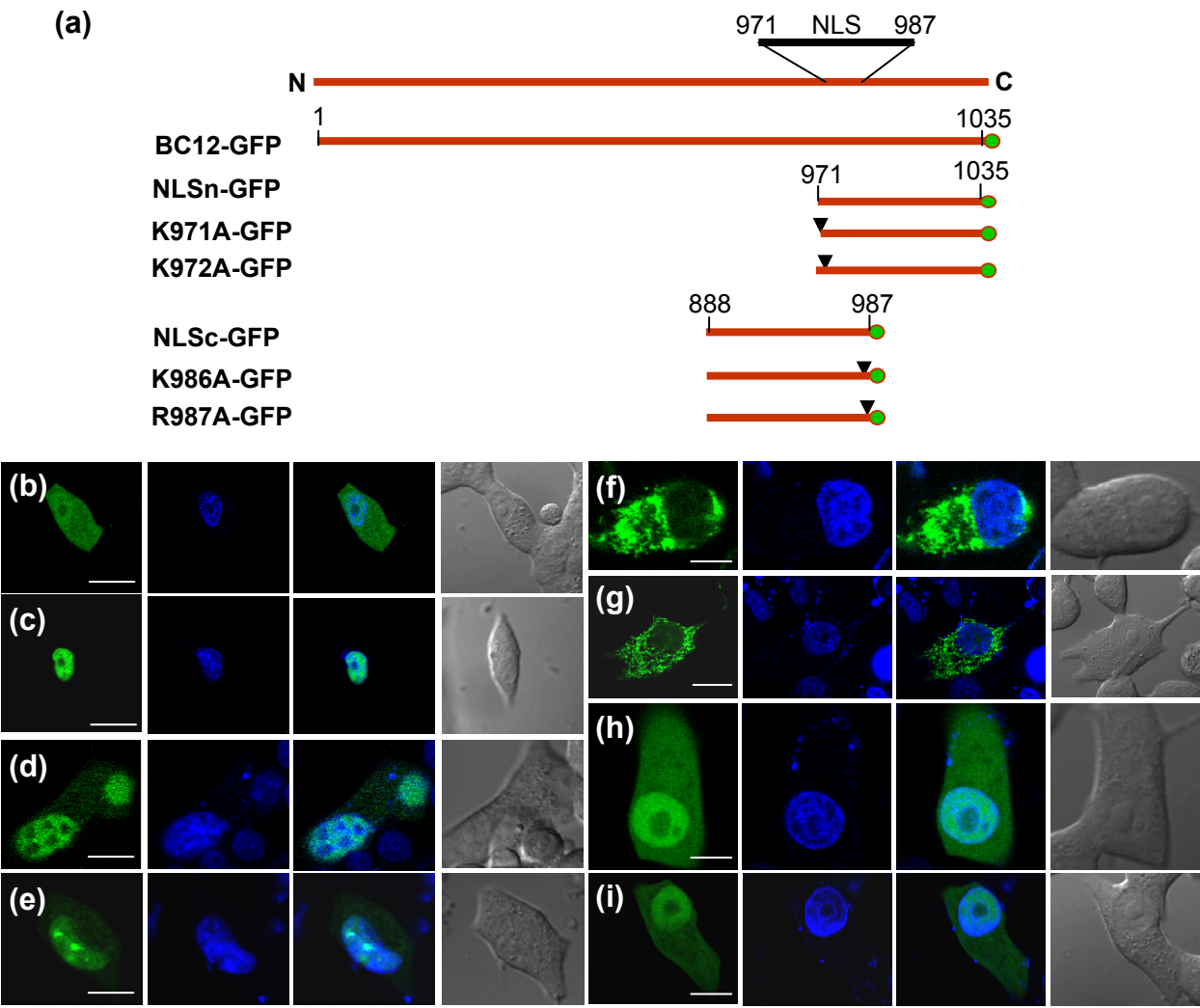

Figure S4

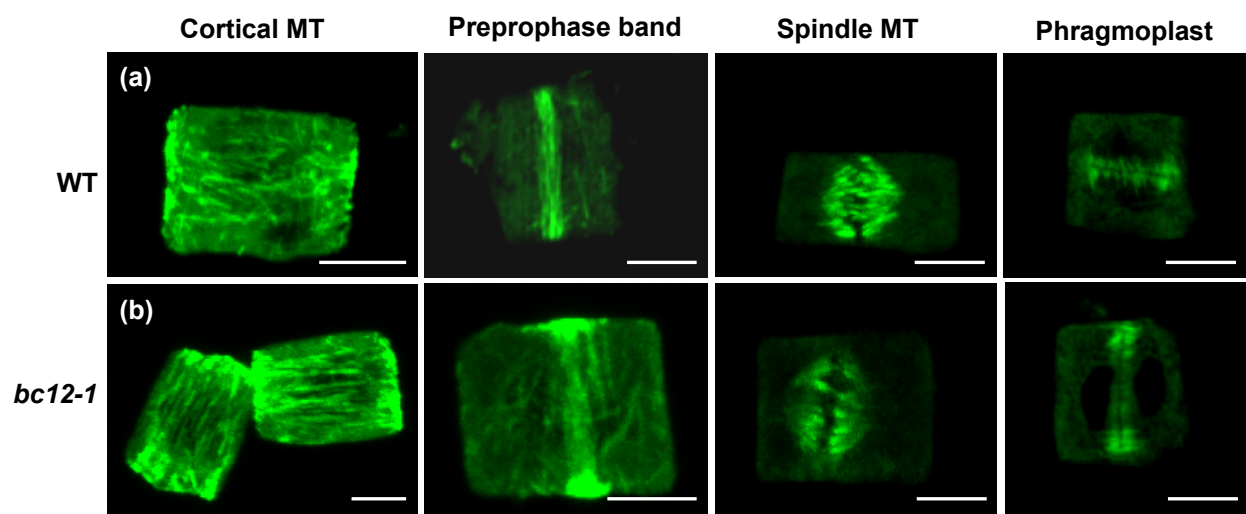

Figure S5

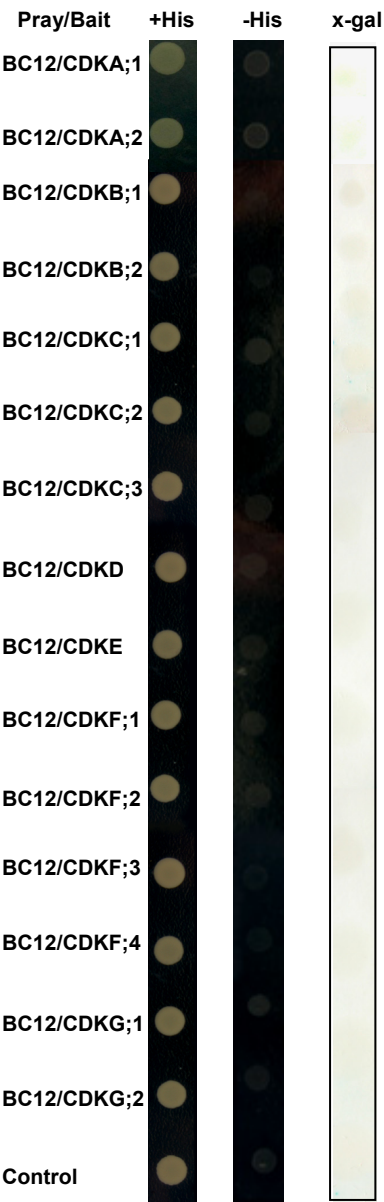

Supplement: Supplementary file 1 [file tpj0063-0312-SD1.pdf]
