# Supplementary material for: Brittle Culm 12, a dual-targeting kinesin-4 protein, controls cell-cycle progression and wall properties in rice
Source: Plant J. 2010 May 26;63(2):312–28. doi: 10.1111/j.1365-313X.2010.04238.x (PMC3440585; doi:10.1111/j.1365-313X.2010.04238.x)
Supplement: Supplementary file 2 [file tpj0063-0312-SD3.doc]

**Supplementary Figure Legends**

**Figure S1.** Characterization of complementary transgenic plants.

(a) A wild-type plant.

(b) A mutant plant overexpressing *BC12* (*BC12OE*), showing the normal height.

(c) A *bc12-1* plant.

(d-f) The culm longitudinal sections of wild-type (d), complementary (*BC12OE*) (e), and *bc12-1* (f) plants, showing the unchanged cell size in these plants.

(g) The measurements of leaf breaking force (mean of 3 replicates ± SD).

(h) Protein gel blotting of the total proteins isolated from *BC12OE*, wild-type (WT), and *bc12-1* plants with anti-BC12 and RbcL antibodies.

Bars = 15 cm in (a-c) and 110 µm in (d-f).

**Figure S2.** Characterization of *BC12* suppression transgenic plants.

(a) A wild-type plant.

(b) A *bc12-2* plant.

(c) A wild-type plant suppression of *BC12* (*BC12RNi*).

(d-f) The culm longitudinal sections of wild-type (d), *bc12-2* (e), and *BC12RNi* (f) plants, showing the unchanged cell size in these plants.

(g) The measurements of leaf breaking force (mean of 3 replicates ± SD).

(h) Protein gel blotting of the total proteins isolated from wild-type (WT), *bc12-2*, and *BC12RNi* plants with anti-BC12 and RbcL antibodies. The arrow heads and a star indicate the normal, reduced size and low-level protein in wild type, *bc12-2*, and *BC12RNi*, respectively.

Bars = 15 cm in (a-c) and 110 µm in (d-f).

**Figure S3.** Identification of the NLS in BC12.

(a) The constructs for identification of NLS.

(b-i) The INS cells transfected with constructs shown in (a). The GFP signal, DAPI stained nucleus, the merged and DIC images are shown sequentially.

(b) A cell transfected with GFP alone, showing equal labeled cytoplasm and nucleus.

(c) A cell transfected with BC12-GFP, showing abundant signals in the nucleus.

(d,e) A cell transfected with NLSn-GFP and NLSc-GFP, respectively, showing abundant signals in the nuclei and some in the cytoplasm.

(f,g) A cell transfected with K971A-GFP (f) and K972A-GFP (g), respectively, showing signals only in the cytoplasm.

(h,i) A cell transfected with K986A-GFP (h) and R987A-GFP (i), respectively, showing signals in the cytoplasm and the nuclei, as is expressing GFP alone (b).

NLS, nuclear localization signal; INS, rat insulinoma cell line.

Bars = 10 µm in (b-i).

**Figure S4.** Comparison of microtubule arrays during cell cycle between the wild-type and *bc12-1* root cells.

(a,b) Immuno-staining MT with anti-α-tubulin antibody in wild-type (a) and mutant (b) root cells, showing morphologically indistinguishable MT arrays in mutant and wild-type cells.

MT, microtubules. Bars = 10 µm in (a) and (b).

**Figure S5.** Yeast two-hybrid assay of BC12 and CDKs from rice genome.

Cotransformants were spotted from an equal cell suspension culture on selective medium (-Leu, -Trp, +His/-His). The results of X-Gal assay are shown at the right.
