# Supplementary material for: Brittle Culm 12, a dual-targeting kinesin-4 protein, controls cell-cycle progression and wall properties in rice
Source: Plant J. 2010 May 26;63(2):312–28. doi: 10.1111/j.1365-313X.2010.04238.x (PMC3440585; doi:10.1111/j.1365-313X.2010.04238.x)
Supplement: Supplementary file 3 [file tpj0063-0312-SD2.doc]

**Supplementary tables**

**Table S1** Molecular markers developed for map-based cloning of *BC12*

| **markers** | **Foward primer** | **Reverse primer** | **Marker type** |
| --- | --- | --- | --- |
| **S6058** | TGCCATGAAGCTTCGTTAGG | TTCGCTGGTGTCCTGCTAG | STS |
| **S5547** | TAAGAGGTAGTGATTGGTGTGAC | CCAGTATTGGCTTTTAAGTCG | STS |
| **S590** | ATGACCGTGGAGGTTGAGTC | CCTACATCCCAACAACATACATAG | STS |
| **S578** | GGTTCCAACACGATACACG | CAAATCGCATACGTGCTCG | STS |
| **S570** | AAGTGACCAAGGGAAGAGC | ACGGCTTCTGTCGTTGTTC | STS |
| **S558** | GGTTCCAACACGATACACG | GCATACGTGCTCGGATTGG | STS |
| **S135** | TGATAGTGCAACGGCAAGG | GTGGAGTTGTCAGCAGTGG | STS |
| **C559** | GAACAACATTAGGATCCTGG | TAAGGACCAACAGCAGGTC | CAPS (*Acc*Ⅰ) |
| **RM444** | GCTCCACCTGCTTAAGCATC | TGAAGACCATGTTCTGCAGG | SSR |
| **RM219** | CGTCGGATGATGTAAAGCCT | CATATCGGCATTCGCCTG | SSR |
| **RM524** | TGAAGAGCAGGAACCGTAGG | TCTGATATCGGTTCCTTCGG | SSR |

**Table S2** The primer sequences used in this study

| **Name** | **Forword Primer** | **Reverse Primer** | **Size (bp)** |
| --- | --- | --- | --- |
| *BC12RNi* | ATGAGCTCGGATCCGTTTCAGCTCGAGGAGGAG | ATACTAGTGGTACCATCAATATCTTTAGGGCTGC | 267 |
| *BC12* | GGATCTCAATGCTTGAACAGAAAA | TCAGGTTCACAAGGATCAGTGTAAC | 91 |
| *cycA1;1* | TAACTACATTGATCGTTATCTTTCTG | TCTCTGAAGTATGTGTTGTCAGTTAT | 158 |
| *cycB2;2* | CTGCATACAAAATACTCTGAAGAAC | TAAATGTGCTGTACTTTCTATGAACT | 173 |
| *cycC* | AGGCACTTGACTATTATTTAGTTGTT | ATGAGAATAAGATCCATCTTGTAAGT | 138 |
| *cycD* | ATAGCTAGAGGAACAGAATGCTTG | GACATCCTCTCCTTATTTACATGG | 118 |
| *CDKA1* | CTGTTCCCTGGTGATTCTGA | TCCAATGTAAAGCCATAGCAG | 301 |
| *CDKA2* | GCCTCATCTTGTCCATTTGT | CCCGCAATAAGGATCTTTCA | 198 |
| *CDKB2* | GAGCATCCCTACTTCAACGA | CAGCATCCAGGAAACAGACA | 164 |
| *KRP1* | CCGCGAGAGGAGAGAAACAA | TGCTGTGCTGAGGCTGTTG | 128 |
| *R2* | CAGAGCGGAACTTCACTCATC | CACTAGAACCCTGGAGAAACG | 158 |
| *E2F1* | TCAGTCGAACGGGAGTAAATCTT | TGCTCCCGTCAAACCAATAACTC | 289 |
| *Ubiquitin5* | ACCACTTCGACCGCCACTACT | ACGCCTAAGCCTGCTGGTT | 69 |
